# Cardiac Arrest as First Presentation of Thyroid Storm

**DOI:** 10.7759/cureus.37057

**Published:** 2023-04-03

**Authors:** Mohamad Zayour, Fatima A Yasmin, Ahmad Baydoun, Michel Tawk, Dana Sleiman, Wassim Shatila, Clara Chamoun

**Affiliations:** 1 Cardiology, University of Balamand, Beirut, LBN; 2 Endocrinology, University of Balamand, Beirut, LBN; 3 Internal Medicine, University of Balamand, Beirut, LBN; 4 Pulmonary and Critical Care, University of Balamand, Beirut, LBN; 5 Endocrinology, Diabetes and Metabolism, University of Balamand, Beirut, LBN

**Keywords:** cardiac arrhythmia, hyperthyroidism, thyrotoxicosis, cardiac arrest, thyroid storm

## Abstract

Thyroid storm is a life-threatening condition characterized by a high level of circulating thyroid hormones and harbors high mortality and morbidity, even if diagnosed and treated early. The condition is frequently overlooked and under-recognized in emergency departments owing to its rarity. Here, we present a case of a 24-year-old male patient, previously healthy, who presented with cardiac arrest and was found to have heart failure and high thyroid hormone levels after investigations. Consequently, the presentation was attributed to thyroid storm. His clinical status and cardiac function improved after treatment of the hyperthyroidism.

## Introduction

Thyrotoxicosis is a familiar endocrine disorder that can have diverse etiologies. Thyrotoxic crisis is considered a critical complication denoted by multiple organ dysfunction. Precisely, the high circulating thyroid hormones have a direct toxic effect on the cardiovascular system. The cardiac manifestations and complications of this condition can include myocardial injury, heart failure, cardiogenic shock, cardiac arrhythmias, and sudden cardiac arrest [1].

To our knowledge, there are few reported cases of cardiac arrest secondary to a thyroid storm [2]. We present here a young patient with a thyroid storm who presented with cardiac arrest.

## Case presentation

A 24-year-old man presented unresponsive to the emergency department. His friends reported that he was sleeping in his bed and woke up shouting incomprehensible words before becoming unresponsive. The patient had been unresponsive for 15 minutes when he arrived at the emergency department. The initial rhythm was pulseless electrical activity and he received immediate cardiopulmonary resuscitation (CPR). He was delivered three shocks for pulseless ventricular tachycardia during the 20 minutes of CPR, following which a return of spontaneous circulation was achieved. The patient was intubated, received vasopressor support, and was transferred to the intensive care unit.

On physical exam, he had no signs of thyroid enlargement, exophthalmos, or jaundice. On auscultation, there was a good bilateral air entry, with no added sounds. The abdomen was soft on palpation. He had no peripheral edema. He had normal cranial and spinal reflexes. He was previously healthy with no significant personal or familial history of thyroid or autoimmune diseases. His colleagues noted severe depression for the past several days and one episode of diarrhea and vomiting on the day of presentation.

After resuscitation, he had low blood pressure (85/67 mmHg), pulse of 150 beats per minute in sinus rhythm, oxygen saturation of 100% while given a fraction of inspired oxygen of 35%, and his temperature was 39.4 degrees Celsius. The laboratory findings revealed normal troponin I, negative C-reactive protein, and normal chemistries. His liver function tests were slightly deranged (Table 1).

**Table 1:** Laboratory findings on admission WBC = white blood cells; HGB = haemoglobin; HCT = haematocrit; BUN = blood urea nitrogen; CRP = C- reactive protein; CPK = creatine phosphokinase; ALAT: alaine amintranferase; ASAT: aspartate aminotransferase.

Test	Result	Normal range
WBC	11.9	4.5 - 11 x10^3^ /μL
HGB	13.7	13 - 16 x 10^6^ g/dL
HCT	38.6	41 - 50 %
Platelets	168	150 - 450 x 10^3^ /μL
Neutrophils	85	55 - 75%
BUN	13	6 - 20 mg/dL
Creatinine	1.04	0.7 - 1.1 mg/dL
Sodium	143	136 - 145 mEq/L
Potassium	3.43	3.5 - 5.1 mEq/L
Chloride	107	90 - 110 mEq/L
Bicarbonate	13	22 - 28 mEq/L
Calcium	8.1	8.6 - 10.2 mg/dL
Magnesium	2.08	1.7 - 2.5 mg/dL
CRP	0.262	< 0.9 mg/dL
Troponin I	0.10	< 0.3 ng/mL
CPK	171	20 - 200 ng/mL
CK-MB	2.4	1.0 - 5.0 ng/mL
ALAT	109	7 – 56 IU/L
ASAT	83	8 – 48 IU/L

**Figure 1:**
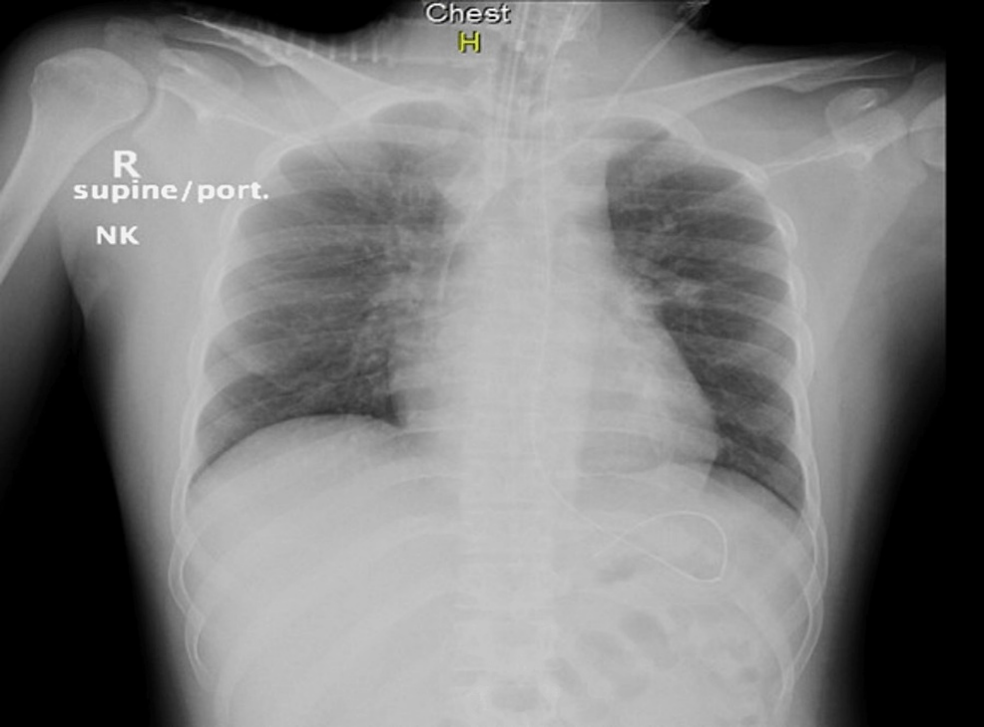
Chest radiograph on admission A nasogastric tube, an endotracheal tube, and a central venous line are seen on the radiograph.

The urine screening was negative for any drugs and medications. Chest radiography did not demonstrate any signs of volume overload, pneumothorax, or infiltrates (Figure 1).

**Figure 2:**
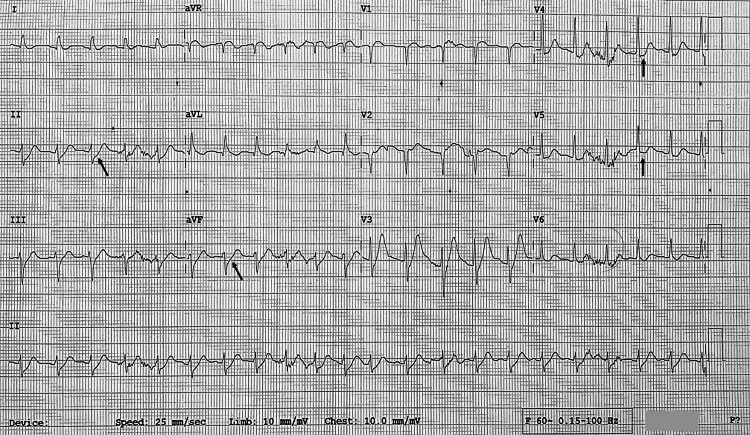
Electrocardiogram on admission Arrows indicate ST segment depression

His electrocardiogram after the return of spontaneous circulation showed a sinus tachycardia with minimal lateral ST depression (Figure 2).

**Figure 3:**
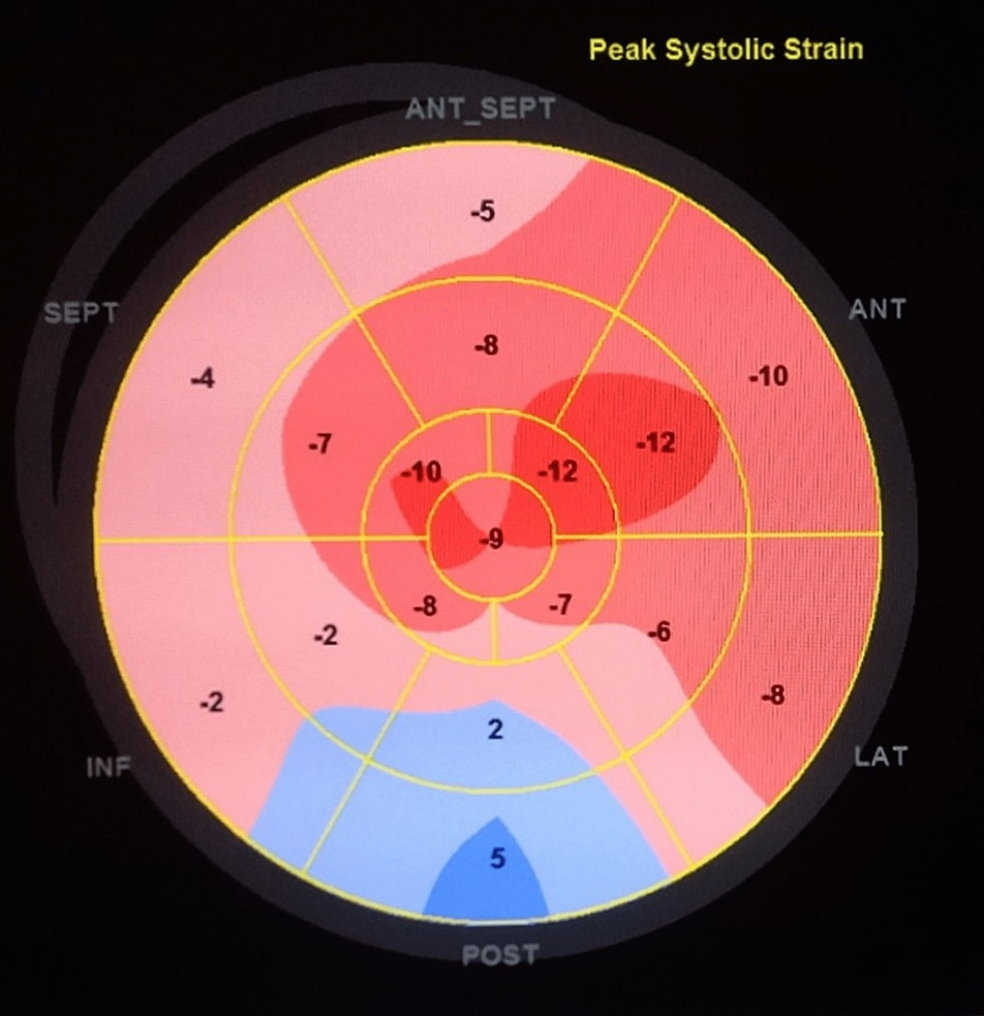
Global longitudinal strain on admission SEPT = septal; ANT_SEPT = antero-septal; ANT = anterior; LAT = lateral; POST = posterior; INF = inferior

An echocardiogram was obtained and indicated severe global hypokinesia with a low left ventricular ejection fraction (LVEF) of 25%, by biplane method and a global longitudinal strain of - 6.6%, as seen in Figure 3, without any sign of cardiac tamponade. Brain computed tomography scan was negative for any bleeding or ischemic changes.

Examination of thyroid function showed a thyroid-stimulating hormone (TSH) level of 0.01 μIU/mL (reference range: 0.27-4.2 μIU/mL), a free thyroxine (T4) level of 2.94 ng/dL (reference range: 0.58-2.3 ng/dL), a free triiodothyronine (T3) level > 20.00 pg/mL (reference range: 1.8-4.6 pg/mL), and absence of TSH receptor antibody (1.11 IU/L). Thyroid ultrasound was unremarkable.

Hyperthyroidism-induced thyroid storm was strongly suspected, and the patient was started on methimazole 20 mg every four hours per nasogastric tube, Lugol’s iodine solution (5%) 10 drops every six hours, and propranolol 20 mg every eight hours as tolerated with respect to his cardiac function.

Repeated cardiac enzymes six hours after the first set showed a troponin I value of 14.93 ng/mL. An acute coronary syndrome treatment was given (aspirin 300 mg, ticagrelor 180 mg, and therapeutic doses of enoxaparin) while awaiting the results of a coronary angiogram.

**Figure 4:**
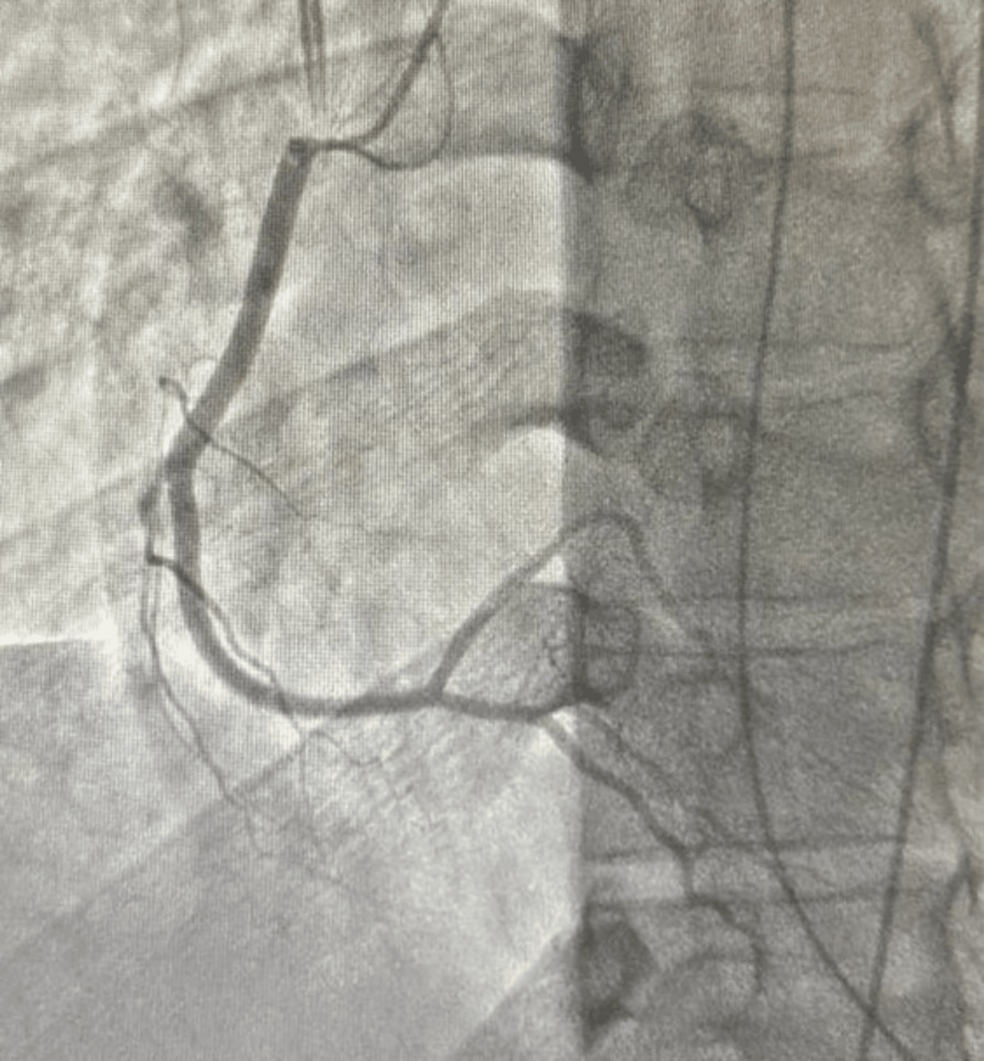
Left anterior oblique cranial view of right coronary artery

**Figure 5:**
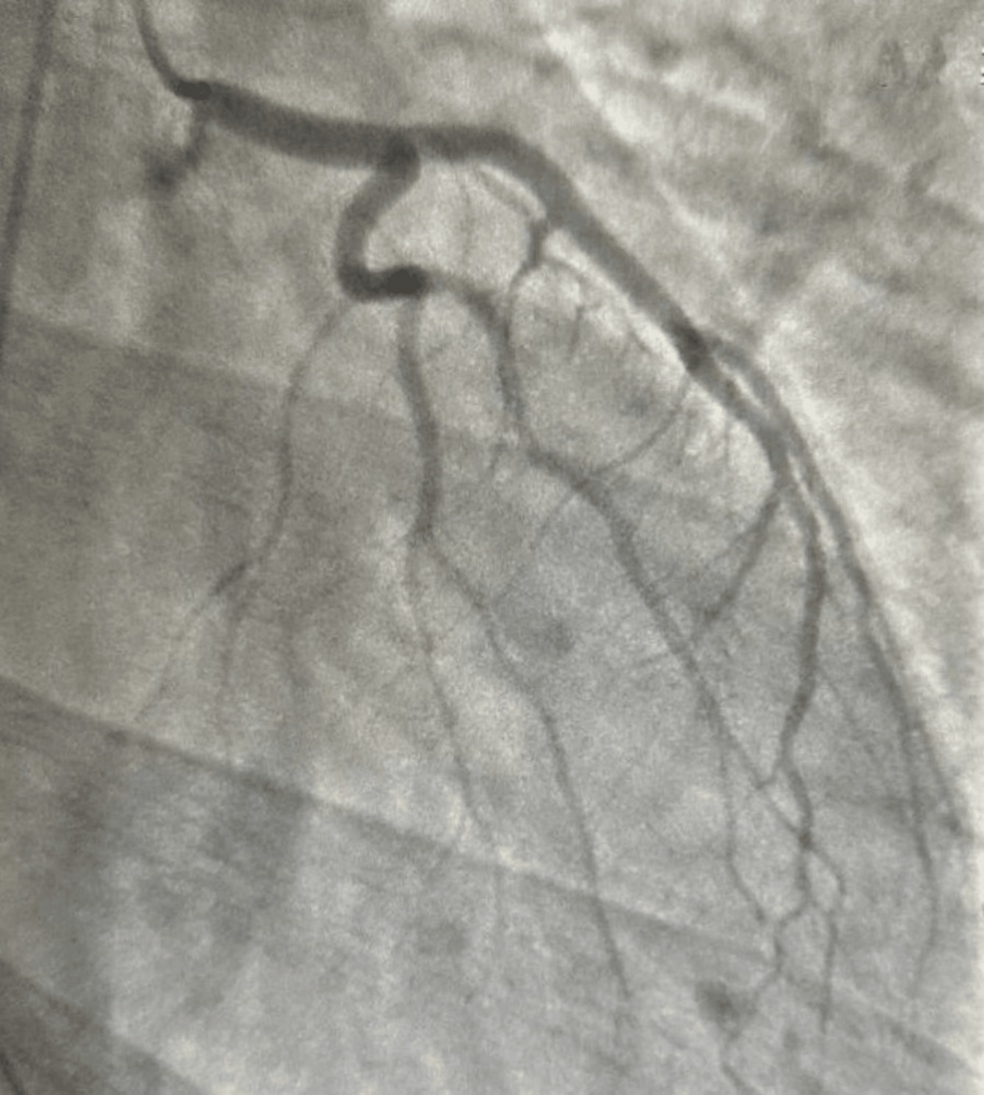
Right anterior oblique caudal view of left coronary system

**Figure 6:**
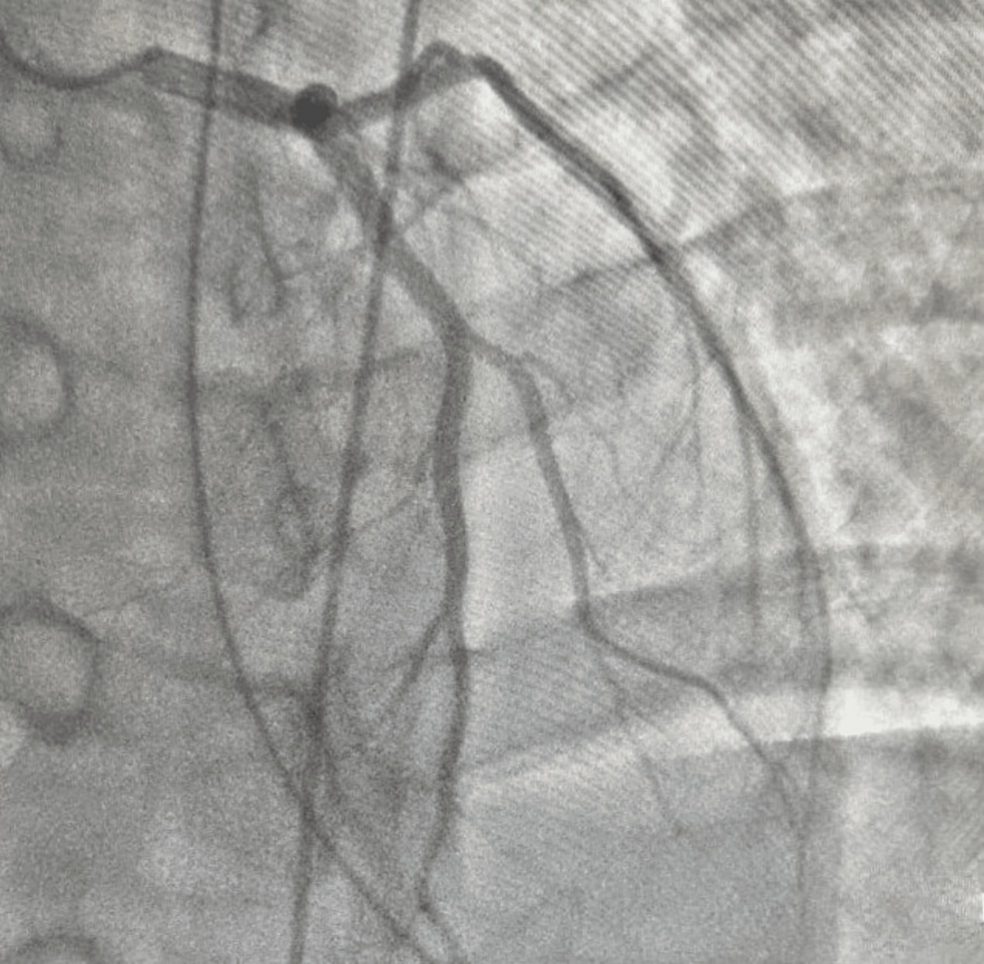
Left anterior oblique cranial view of left coronary system

On day 2 of hospitalization, cardiac catheterization was done (Figures 4-6) with no evidence of coronary artery disease. The acute coronary syndrome treatment was then stopped.

**Figure 7:**
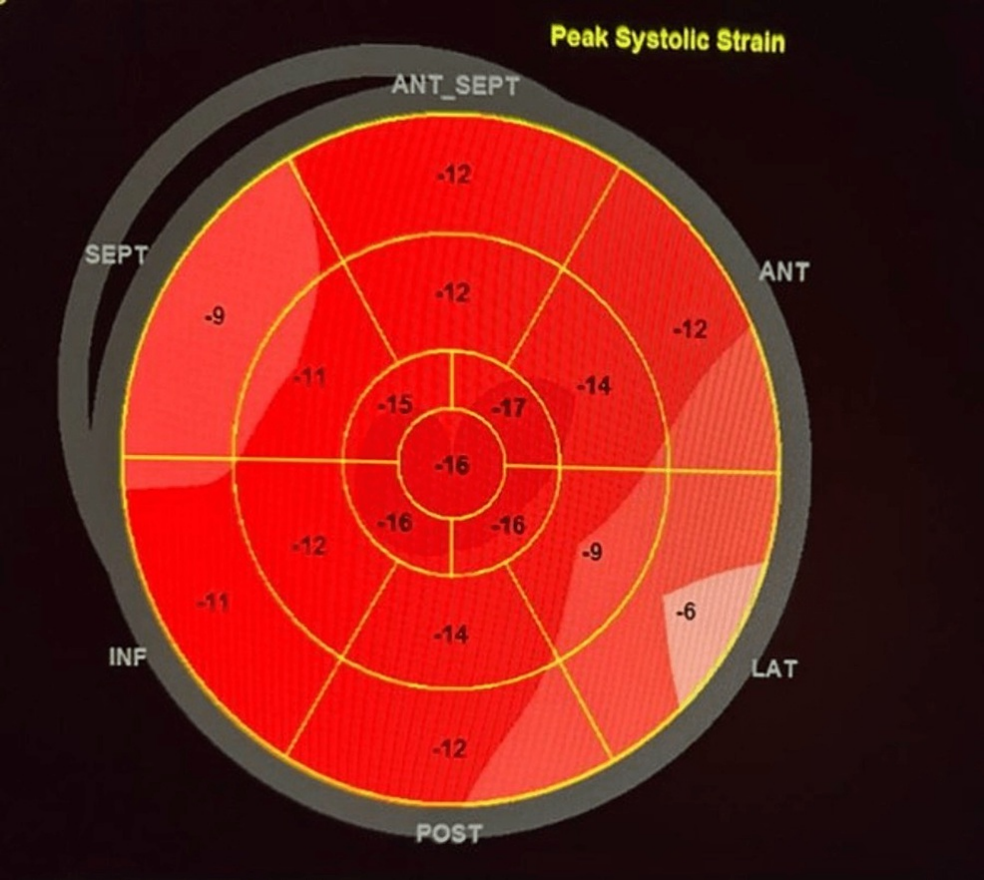
Global longitudinal strain on day 3 of hospitalization SEPT = septal; ANT_SEPT = antero-septal; ANT = anterior; LAT = lateral; POST = posterior; INF = inferior

On day 3 of hospitalization, vasopressors were stopped, with vital signs within the normal range. Repeated echocardiography showed improved LVEF of 50-55% calculated by biplane method with global longitudinal strain of -12.6% as shown in Figure 7.

The dosage of methimazole was tapered to 20 mg every six hours per nasogastric tube. At this time, hydrocortisone was started (300 mg as loading dose then 100 mg every eight hours). Hydrocortisone initiation was delayed until a diagnosis of myocarditis was excluded. On day 5 of hospitalization, the patient was weaned from the respirator. An electroencephalogram demonstrated diffuse slowing, but no subclinical or clinical seizures. Extubation was not possible due to his neurologic state, so a tracheostomy tube was placed on day 10 of hospitalization. Frequent thyroid panels, during his hospital stay, are shown in Table 2.

**Table 2:** Thyroid function tests and troponin evolution during hospitalization TSH = thyroid stimulating hormone; TRAB = TSH receptor antibody

Test	Day 1 (3am)	Day 1 (8am)	Day 1 (5pm)	Day 2	Day 3	Day 4	Day 5	Day 6	Day 30	Normal range
Troponin I	0.10	14.93	-	-	0.97	0.46	0.18	-	-	<0.3 ng/mL
TSH	0.01	-	-	-	-	-	< 0.01	-	2.1	0.24-4.2uIU/ml
Free T3	-	> 20.0	8.54	4.98	2.00	-	-	< 1.5	1.22	1.8-4.6 pg/mL
Free T4	-	2.94	3.09	2.85	1.88	-	1.18	1.06	1.33	0.58-2.3 ng/dL
Total T3	-	-	-	-	-	-	< 0.4	-	-	0.8-2 ng/ml
TRAB	Negative									

**Figure 8:**
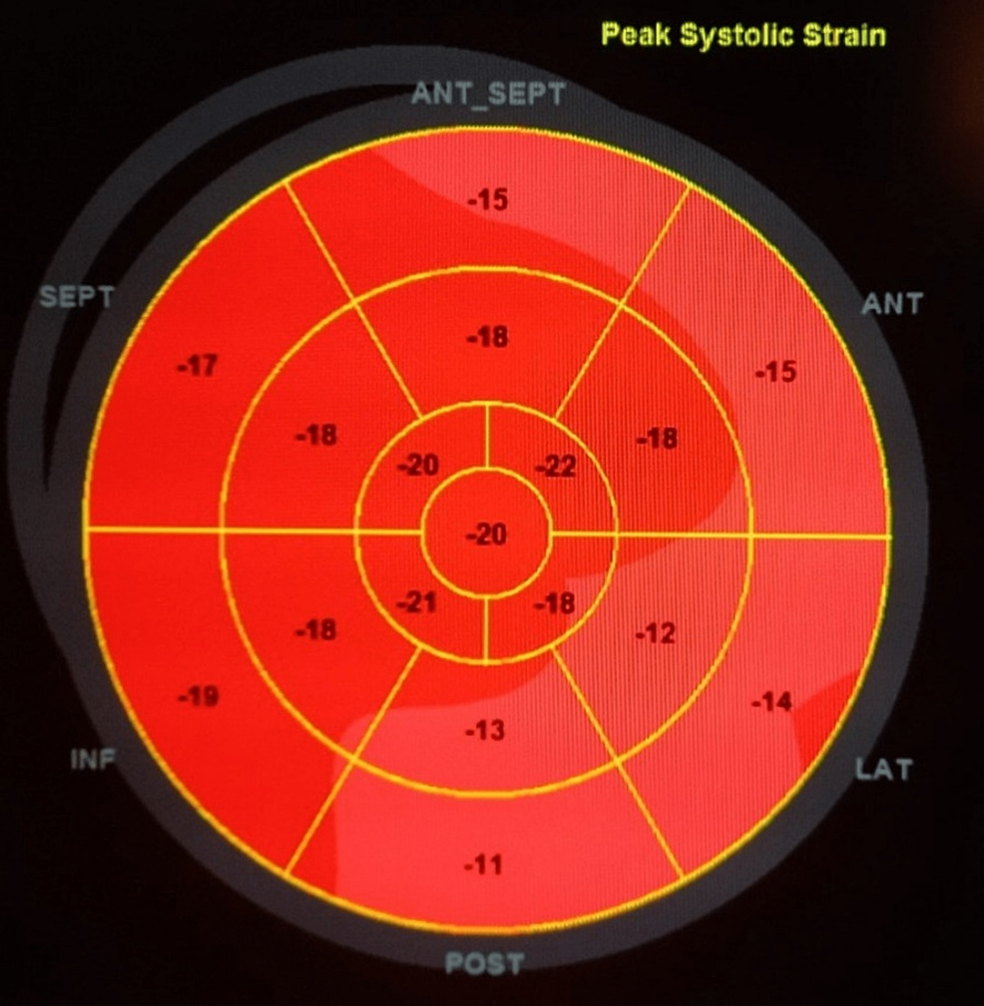
Global longitudinal strain on day 30 of hospitalization SEPT = septal; ANT_SEPT = antero-septal; ANT = anterior; LAT = lateral; POST = posterior; INF = inferior

On day 30 of hospitalization, follow-up testing revealed that free T4, free T3, and TSH levels had returned to normal (free T4 level: 1.33 ng/dL, free T3 level: 1.22 pg/ml, and TSH level: 2.1 μIU /dL). Echocardiography was also performed again and showed a normalization of the systolic heart function with global longitudinal strain of -17% (Figure 8).

The patient was at this point doing well. The tracheostomy tube was removed, and he had normal motor power in all his limbs and could speak and understand language; however, he had some cognitive impairment.

## Discussion

In the present case, a previously healthy young man presented with cardiac arrest and disturbed thyroid function tests. He had severe heart failure with no obstructive coronary artery disease. He had high-grade fever with negative inflammatory markers and no focus of infection causing the hyperthermia. Drug intoxication was ruled out. There was no reason for his cardiac arrest except a severe hyperthyroid state. The treatment of thyrotoxicosis resulted in complete recovery of the cardiac function with normal hemodynamics. Therefore, with a negative workup for other causes of cardiac arrest, a likely trigger here was the hyperadrenergic effect of a thyroid storm. The thyrotoxicosis is presumed to be from increased synthesis, since a methimazole dose of 5 mg every eight hours is still required, many weeks after the presentation, to maintain a normal thyroid hormones level.

A thyroid storm is a condition characterized by the severe clinical manifestation of thyrotoxicosis. It is a life-threatening condition with a high mortality rate, even if detected early and treated properly [3,4]. The manifestations are mainly due to hypermetabolism, which is secondary to high circulating thyroid hormones [5].

This increase in thyroid hormones can be due to increased synthesis as in Grave’s disease, thyroid adenomas, toxic multinodular goiter, an exogenous intake of thyroid hormones, or an abnormal release as in thyroiditis [6].

The clinical presentation includes mental status changes (agitation, anxiety, delirium, psychosis, stupor, or coma), gastrointestinal symptoms, cardiac arrhythmia, hypo or hypertension, congestive heart failure, shock, and death [7]. A retrospective multicenter study conducted by Bourcier et al. found that the most frequent thyroid storm clinical manifestations were congestive heart failure (72%), central nervous system impairment (63%), and gastrointestinal or hepatic manifestations (52%) [4]. Fifteen percent of the patients had cardiac arrest before admission to the ICU. Sixty percent had supraventricular tachycardia and 13% had ventricular fibrillation [4].

The Burch and Wartofsky scoring system is used for the diagnosis of thyroid storms [8]. Our patient’s score on admission was more than 45 points (25 points for thermoregulatory dysfunction, 25 points for cardiovascular dysfunction, 30 points for central nervous system effects, and 10 points for gastrointestinal-hepatic dysfunction). This further supports the diagnosis of thyroid storm in our patient. 

Thyroid hormones modulate every component of the cardiovascular system necessary for normal cardiovascular development and function. The major effects on the heart are mediated by T3, which increases the force and speed of contraction and the speed of relaxation [9]. Cardiopulmonary failure is the most common cause of death in thyroid storm, especially in the elderly [10].

Many of the clinical manifestations of thyrotoxicosis are due to hormonal effects on hemodynamics [11]. A thyroid storm can cause acute myocardial infarction, coronary vasospasm, stress cardiomyopathy, autoimmune myocarditis, and ventricular arrhythmias [1]. In this case, we think that a ventricular arrhythmia caused the cardiac arrest of the patient.

The pathophysiology of heart failure induced by thyrotoxicosis is poorly understood but potentially reversible and curable; this is why the condition should be excluded in every new patient with heart failure, especially in the absence of coronary artery disease and other structural heart diseases [12]. Myocardial damage, secondary to the toxic effect of high serum-free thyroid hormones leads to alterations in energy production by the cardiomyocyte (oxidative phosphorylation glycolysis), intracellular metabolism (protein synthesis), and myofibril contractile function. Cardiac manifestations include left ventricular hypertrophy, tachycardia, atrial fibrillation, dilated cardiac chambers, pulmonary hypertension, and diastolic dysfunction [12-14].

The morbidity and mortality are high but the prognosis is good since the function is restored with the correction of the hyperthyroid state [13]. In a study of 591 patients who presented with primary hyperthyroidism, 5.8% were found to have congestive heart failure as the first presentation, and half of them had left ventricular systolic dysfunction. Improvement of symptoms and ejection fraction was detected after the establishment of a euthyroid state. However, one-third had persistent dilated cardiomyopathy [12]. In our patient’s case, the systolic function returned to normal after treatment of the hyperthyroid state.

## Conclusions

We describe the case of a young patient who presented for cardiac arrest, and was found to have severe heart failure; after a thorough examination, the only abnormality detected was the high thyroid hormone levels. The overall status improved after the treatment of hyperthyroidism. A diagnosis of thyroid storm was made. Hence, thyroid storm and thyroid dysfunction should be kept in the differential diagnosis in patients presenting with cardiac arrest or heart failure after ruling out more common causes.

## References

[1] Brown J, Cham MD, Huang GS (2020). Storm and STEMI: a case report of unexpected cardiac complications of thyrotoxicosis. Eur Heart J Case Rep.

[2] Nakashima Y, Kenzaka T, Okayama M, Kajii E (2014). A case of thyroid storm with cardiac arrest. Int Med Case Rep J.

[3] Sarlis NJ, Gourgiotis L (2003). Thyroid emergencies. Rev Endocr Metab Disord.

[4] Bourcier S, Coutrot M, Kimmoun A (2020). Thyroid storm in the ICU: a retrospective multicenter study. Crit Care Med.

[5] Kopp P (2010). Thyrotoxicosis of other etiologies. Endotext [Internet].

[6] Reinwein D, Benker G, König MP, Pinchera A, Schatz H, Schleusener A (1988). The different types of hyperthyroidism in Europe. Results of a prospective survey of 924 patients. J Endocrinol Invest.

[7] Ngo SY, Chew HC (2007). When the storm passes unnoticed--a case series of thyroid storm. Resuscitation.

[8] Carroll R, Matfin G (2010). Endocrine and metabolic emergencies: thyroid storm. Ther Adv Endocrinol Metab.

[9] Grais IM, Sowers JR (2014). Thyroid and the heart. Am J Med.

[10] Scholz GH, Hagemann E, Arkenau C (2003). Is there a place for thyroidectomy in older patients with thyrotoxic storm and cardiorespiratory failure?. Thyroid.

[11] Klein I, Ojamaa K (2001). Thyroid hormone and the cardiovascular system. N Engl J Med.

[12] Siu CW, Yeung CY, Lau CP, Kung AW, Tse HF (2007). Incidence, clinical characteristics and outcome of congestive heart failure as the initial presentation in patients with primary hyperthyroidism. Heart.

[13] Babenko AY, Bairamov AA, Grineva EN, Ulupova EO (2012). Thyrotoxic cardiomyopathy. Basic Research to Clinical Management.

[14] Albakri A (2018). Thyrotoxic heart failure: a review of clinical status and meta-analysis of electrocardiogram diagnosis and medical clinical management methods. Integr Mol Med.

